# Impact of genital warts on health related quality of life in men and women in mainland China: a multicenter hospital-based cross-sectional study

**DOI:** 10.1186/1471-2458-12-153

**Published:** 2012-03-02

**Authors:** Ju-Fang Shi, Dian-Ju Kang, Shu-Zhen Qi, Hai-Yan Wu, Yan-Chun Liu, Li-Jun Sun, Li Li, Ying Yang, Qing Li, Xiang-Xian Feng, Li-Qin Zhang, Jie Li, Xiao-Li Li, Yun Yang, Mayinuer Niyazi, Ai-Di Xu, Jia-Hua Liu, Qing Xiao, Lian-Kun Li, Xin-Zheng Wang, You-Lin Qiao

**Affiliations:** 1 Department of Cancer Epidemiology, Cancer Institute/Hospital, Chinese Academy of Medical Sciences, Peking Union Medical College, 17 South Panjiayuan LN, PO Box 2258, Beijing 100021, China; 2 Cancer Epidemiology Research Unit, Cancer Council NSW, New South Wales, Australia; 3 Sichuan Center for Disease Control and Prevention, Chengdu, China; 4 Institute of Dermatology, Chinese Academy of Medical Sciences, Peking Union Medical College, Nanjing, China; 5 202 Hospital of Chinese PLA, Shenyang, China; 6 Beijing Ditan Hospital, Beijing, China; 7 Beijing You'an Hospital, Beijing, China; 8 Daping Hospital, Third Military Medical University of Chinese PLA, Chongqing, China; 9 Tangdu Hospital, Fourth Military Medical University of Chinese PLA, Xi'an, China; 10 Shenzhen Women and Children Hospital, Shenzhen, China; 11 Changzhi Medical College, Changzhi, China; 12 General Hospital of Tianjin Medical University, Tianjin, China; 13 Hunan Provincial People's Hospital, Changsha, China; 14 Xinmi Women and Children Hospital, Xinmi, China; 15 Medical School of Yichun University, Yichun, China; 16 People's Hospital of Xinjiang Uygur Autonomous Region, Urumchi, China; 17 Hongkou District Bureau of Health, Shanghai, China; 18 Fujian Provincial Hospital, Fuzhou, China; 19 Women and Children Hospital of Guangzhou City, Guangzhou, China; 20 Liaoning Cancer Hospital, Shenyang, China; 21 The 2nd People's Hospital of Yangcheng County, Shanxi, China

## Abstract

**Background:**

Information on the health-related quality of life (HRQoL) of patients with genital warts (GW) in populations in mainland China is still limited. The aim of the study was to use a generic instrument to measure the impact of genital warts on HRQoL in men and women in this setting.

**Methods:**

A multi-centre hospital-based cross-sectional study across 18 centers in China was conducted to interview patients using the European quality of life-5 dimension (EQ-5D) instrument; respondents' demographic and clinical data were also collected.

**Results:**

A total of 1,358 GW patients (612 men, 746 women) were included in the analysis, with a mean age of 32.0 ± 10.6 years. 56.4% of the patients reported some problems in the dimension of Anxiety/Depression (highest), followed by Pain/Discomfort (24.7%) and Mobility (3.5%). The overall visual analogue scale (VAS) score of the study population was found to be 65.2 ± 22.0, and the EQ-5D index score was found to be 0.843 ± 0.129 using Japanese preference weights (the Chinese preference was unavailable yet). Patients with lower VAS means and EQ-5D index scores were more often female, living in urban area, and suffering multiple GW (all p values < 0.05), but the values did not differ notably by age (p values > 0.05).

**Conclusions:**

The HRQoL of patients with GW was substantially lower, compared to a national representative general population in China (VAS = ~80); the findings of different subgroups are informative for future GW prevention and control efforts.

## Background

Genital warts (GW) is one of the most common sexually transmitted diseases and about 90% of GW are caused by infection with human papillomavirus (HPV)-6 and HPV-11 [1]. The incidence of GW is increasing globally although some developed countries, such as US and Australia, have reported relatively low rates ( < 0.3%) [2,3]. Information on the disease burden of GW in mainland China is still limited, but a relatively higher GW prevalence (0.5%) was reported in general populations of other Asian regions (including India, Taiwan and Hong Kong) [1]. However, the variability in the GW prevalence among different data sources from other Asia Pacific countries might suggest that under-reporting potentially existed in the population surveillance systems [1]. In addition, a high post-treatment recurrence rate of GW has been reported (40%) [4] and this characteristic of GW imposes a considerable sociopsychological burden (such as worries and concerns, negative emotional and sexual impact, shame or stigma, and worse interactions with partner and/or doctors [5,6]) and economic burden [7] on the patients.

It has been estimated that multivalent prophylactic HPV vaccine has the potential to protect up to 83% of genital warts or condylomata acuminate in mainland China [8]. In an era of HPV vaccination, a number of cost-effectiveness analyses (CEA) on multivalent HPV vaccines have been performed globally. Health related quality of life (HRQoL) contains self-reported measures of physical and psychological aspects, and an accurate impact analysis of genital warts on HRQoL was expected to contribute to the health economic evaluation on (HPV) vaccines protects against related HPV types. The extent of HRQoL of patients with GW has previously been quantified in different settings using generic instruments [6,9-11], including the European quality of life-5 dimension (EQ-5D) measure. EQ-5D is a standardized and commonly used instrument on HRQoL [12], and is also one of only a few measures recommended for use in cost-effectiveness analysis [13,14]. However, most of the quality of life studies using EQ-5D in China's populations were more focused on the general populations or patients with various chronic diseases [15-18], and assessment related to GW in this setting has not been performed.

The Cancer Foundation of China (CFC) and the Cancer Institute of Chinese Academy of Medical Sciences (CICAMS) jointly conducted a multi-centre hospital-based epidemiological and economic study on genital warts across China. As part of this broad project, the current study's aim was to use the generic instrument EQ-5D to measure the impact of genital warts on health related quality of life in men and women in mainland China.

## Methods

**Figure 1:**
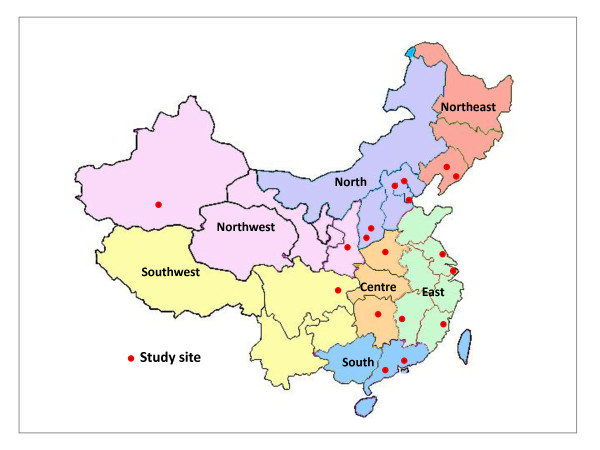
**The distribution of study sites in China**. The 11 higher level centers included Beijing (2 centers), Tianjin, Shenyang (Liaoning Province), Xi'an (Shaaxi Province), Changsha (Hunan Province), Chongqing, Shenzhen (Guangdong Province), Shanghai, Nanjing (Jiangsu Province) and Fuzhou (Fujian Province). The seven lower level centers included Dashiqiao County (Liaoning Province), Hetian County (Xinjiang Autonomous Region), Xinmi Prefecture (county-level, Henan Province), Panyu/Conghua County (Guangdong Province), Tonggu County (Jiangxi Province), Yangcheng County and Changzhi Prefecture (Shanxi Province). Data related to the less-developed centre in Southwest China were not available. Most of the study hospitals have had well established collaboration with CICAMS on a number of prior studies associated with HPV and cervical cancer.

From July 2007 to July 2008, patients with genital warts were enrolled in 18 selected centers across seven geographic regions of China (Northeast, North, Northwest, Central, Southwest, South and East China). Generally, within each region, one province-level centre in a more developed area, and one county-level centre in a less developed area were selected. In addition, four additional study sites were selected from the North and East China regions in order to provide more information on more developed areas or metropolitan cities. Thus, a total of 11 higher level centers and 7 lower level centers eventually participated in the current survey (Figure 1).

A total of 100 GW patients were initially required for each centre at a higher level and 50 patients at a lower level, and a convenience sampling method was applied within each centre. Patients who were currently diagnosed with genital warts, aged 18 years or older and provided informed consent (no participation fee applied) were eligible for this study regardless of their gender or whether they were an incident or recurrent case. Central institutional review board approval was obtained from the Cancer Foundation of China.

The EQ-5D instrument has several language versions and has been applied to a wide variety of populations and diseases [12], and promising evidence for the measurement equivalence of EQ-5D's English and Chinese versions has been reported [19]. EQ-5D contains five dimensions measurement, including Mobility, Self-care, Activities, Pain/Discomfort and Anxiety/Depression [12]. Three options are listed for each dimension: whether the subject has no problem, a moderate problem or an extreme problem. Separately, EQ-5D instrument also contains a visual analogue scale (VAS) which allows the study subjects themselves to assess and self-record their health status from 0 (the worst) to 100 (the best) [12]. Using the EQ-5D instrument (Chinese version), a face-to-face interview was administrated by a project staff member, or alternatively a capable patient completed the questionnaire by him or herself in the presence of a trained interviewer who answered any questions the participants had about the questionnaire. In addition, further patient information including place of residence, education, income, marriage status, whether or not the patient is a smoker, sexual behavior and clinical characteristics were also collected. Information from patients who did not accept the invitation was not recorded in the study.

Prior to the larger-scale survey, a pilot study was conducted in three sites (urban: Beijing; rural: Shanxi and Jiangxi); the initial study flowchart and questionnaire contents (excluding the EQ-5D questionnaire) were then modified and optimized based on the feedback from the pilot study. An investigators' meeting was then organized by CICAMS and CFC to provide data collection training and to introduce the study protocol and an operations manual to all the co-principle investigators and the main project members from 18 sites. The meeting participants were then required to provide training to other project team members (including nurses and physicians at the clinics) in local individual centers, based on the study operation manual. The operations manual contained questionnaire instructions, coding, quality control process, collaborators' responsibility, logistic instructions and other details in an operational level. CICAMS was responsible for all database building, logistical data checks (by interacting with local staff) and data analysis. Data collection and double entry and basic data checks were conducted in the 18 collaborative centers/hospitals.

The characteristics of the study population were described in general terms. Percentages of patients reporting any problems, age-specific means of EQ-5D VAS scores and their 95% confidence intervals were analyzed (overall and by sex), using Chi-square test or one-way ANOVA approach. Because of the absence of a EQ-5D preference weight set for the Chinese population, we applied the preference weight sets from other populations (the UK, US and Japan) [20-22] to estimate the mean EQ-5D index score of this GW population in China. Detailed VAS means and EQ-5D index scores by population characteristics were calculated and a one-way ANOVA approach was applied to detect the statistical differences between subgroups. In addition, Pearson correlation among VAS means and EQ-5D index scores using the three preference weight sets were characterized using a univariate linear regression model. Since a very high association between weighted and non-weighted EQ scores has been reported [23], we then conducted a multivariate linear regression analysis to check the robustness of important variables, by inputting variables with statistical significance confirmed in the non-weighted analysis. For the ordinary unordered categorical variables with more than two subgroups, dummy variables were applied and the subgroup with lowest mean/score (which was southwest in the region category and recurrent GW in the clinical status category) was chosen as the reference subgroup. All analysis used two tailed tests.

## Results

A total of 1,358 outpatients with genital warts were included in the analysis (612 men and 746 women), with a mean age of 32.0 years (SD: 10.6, range: 18-86). Of all study patients, 75.1% of them were enrolled from urban-based hospitals, 33.2% received college or higher education, 30.0% held health insurance, 22.2% were current frequent smokers, and their median monthly income was 1,400 Chinese Yuan (~200 US$). In addition, 69.3% of the study subjects were married or living with their partners, and 54.3% have had two or more lifetime sexual partners. During the survey, the patients were basically categorized into three clinical statuses, including the initial clinical visit for GW, the follow-up visit within an episode, and visits for recurrent GW, with the proportions in each status being 76.0%, 10.8% and 13.3%, respectively. In all, 26.2% of the subjects were observed to have a single genital wart on their bodies, while the rest suffered multiple genital warts.

Table 1 illustrates the results of the five study dimensions. For the whole study population, the dimension of Anxiety/Depression had the highest rate of self-reported problems (56.4%), followed by Pain/Discomfort (24.7%), Mobility (3.5%), Usual activities (2.8%) and Self-care (0.5%). Compared to the male patients, females tended to report more problems in three dimensions, including Anxiety/Depression (50.1% vs. 61.5%), Pain/Discomfort (20.6% vs. 28.0%), and Mobility (1.3% vs. 5.2%) (all *P *values < 0.005). The overall VAS mean among all cases was 65.2 (SD: 22.0), and men had a higher mean score than women (69.0 vs. 62.1, *P *< 0.001). To make comparisons for all of the reported groups, relevant findings from a national population-based survey in China [15] were also added to Table 1 as references. After applying the preference weights of the Japanese, UK and US populations, the overall EQ-5D index scores of the current analysis were 0.843, 0.826 and 0.859, respectively; and similarly, men scored higher than women under every one of the three preference weight sets (all *P *values < 0.001) (Table 2).

**Table 1:** Percentages of subjects self-reported problems and means of visual analogue scale (VAS), total and by sex


**EQ-5D dimension**	**The current study**						**Findings from a national population-based survey in China, 2008 **[15]		
	
	**Male**		**Female**		**Total**		**Male**	**Female**	Total*
	
	**No**.	**%**	**No**.	**%**	**No**.	**%**	**% (N = 58,163)**	**% (N = 62,540)**	**% (N = 120,703)**

Mobility									

No	604	98.7	707	94.8	1,311	96.5	95.7	94.6	95.1

Moderate	8	1.3	38	5.1	46	3.4	4.0	5.0	4.5

Extreme	0	0.0	1	0.1	1	0.1	0.3	0.4	0.4

Self-care									

No	610	99.7	741	99.3	1,351	99.5	97.3	96.6	96.9

Moderate	2	0.3	5	0.7	7	0.5	2.3	3.0	2.7

Extreme	0	0.0	0	0.0	0	0.0	0.4	0.4	0.4

Usual activities									

No	596	97.4	723	96.9	1,319	97.1	96.0	94.9	95.4

Moderate	16	2.6	21	2.8	37	2.7	3.3	4.3	3.8

Extreme	0	0.0	2	0.3	2	0.1	0.7	0.8	0.8

Pain/Discomfort									

No	486	79.4	537	72.0	1,023	75.3	92.8	89.5	91.1

Moderate	122	19.9	205	27.5	327	24.1	6.9	10.0	8.5

Extreme	4	0.7	4	0.5	8	0.6	0.3	0.5	0.4

Anxiety/Depression									

No	305	49.8	287	38.5	592	43.6	94.8	92.9	93.8

Moderate	259	42.3	362	48.5	621	45.7	4.9	6.7	5.8

Extreme	48	7.8	97	13.0	145	10.7	0.3	0.4	0.4

VAS score									

	612	69.0 (mean)	746	62.1 (mean)	1,358	65.2 (mean)	80.9 (mean)	79.4 (mean)	80.1 (mean)

Chi-square tests were performed, female patients tended to report more problems in three dimensions, including Anxiety/Depression, Pain/Discomfort and Mobility, when comparing to male patients (all *P *values < 0.005).

* The percentages of "Total" group were estimated based on the reported findings for males and females with considering each group's sample size.

**Table 2:** The EQ-5D index score using different preference weights, total and by sex


**Preference weight set applied**	**Male**		**Female**		**Total**	
	
	**Mean**	**SD**	**Mean**	**SD**	**Mean**	**SD**

Japan	0.864	0.13	0.827	0.126	0.843	0.129

UK	0.856	0.185	0.802	0.21	0.826	0.201

US	0.882	0.137	0.84	0.15	0.859	0.145

One-way ANOVA approach was applied to compare scores between men and women

(all *P *values < 0.001)

A range of strengths of correlation between results from different analyses were characterized. On one hand, relatively low Pearson correlations R between EQ VAS mean and EQ-5D index scores were observed (0.371, 0.423, 0.412 using Japanese, UK and US preference weights respectively, all *p *values < 0.001). On the other hand, unsurprisingly, a slightly higher agreement (0.984, *p *< 0.001) was observed between EQ-5D index scores of UK and US preference weights compared to those of the other two pairs (Japan & UK: 0.817, Japan & US: 0.860, all *p *values < 0.001); this situation is potentially due to the cultural dissimilarity between Western and Asian populations. In addition to this, compared to the EQ-5D results for populations in the UK and US, the reported percentages of subjects' problems by each of the EQ-5D's five dimensions among the Japanese general population were closer to those of the general population in China [15,17]. For this reason, only the preference weight set of the Japanese population was applied when we calculated further characteristic-specific EQ-5D index scores in this analysis.

**Figure 2:**
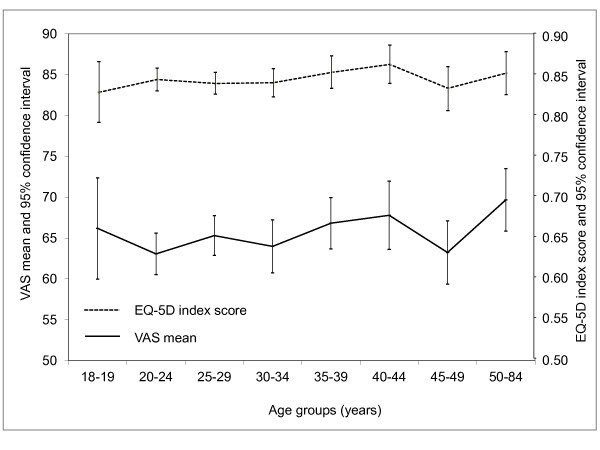
Age-specific means of visual analogue scale (VAS) and EQ-5D index score*. One-way ANOVA analyses were performed (*P *> 0.05); * Japanese preference weights applied.

Table 3 presents further detailed mean VAS and EQ-5D index scores for different subgroups. Briefly, female patients, living in urban areas, residing in the Southwest region, smoking less frequently and with multiple clinically-confirmed GW were statistically associated with lower VAS and EQ-5D index scores (one-way ANOVA results, all *p *values < 0.05); and a lower monthly income and the situation of recurrent GW probably contributed to a worse HRQoL of GW patients (*P *< 0.05 in the EQ-5D index score calculation, but *P *> 0.05 in the VAS analysis). Significant differences were not detected among subgroups by age (Figure 2), marital status, education, insurance or the number of lifetime sexual partners (all *P *values > 0.05).

**Table 3:** Mean visual analogue scale (VAS) and EQ-5D index score, by population characteristics*


**Variable**	**No**.	**%**	**VAS**			EQ-5D index score**		
			
			**Mean**	**SD**	**Sig.^#^**	**Mean**	**SD**	**Sig.^#^**

Sex								
Male	612	45.1	69.0	21.3	< 0.001	0.864	0.130	< 0.001
Female	746	54.9	62.1	22.1		0.827	0.126	
Region								
North	372	27.4	66.6	25.0	< 0.001	0.866	0.135	< 0.001
Northeast	128	9.4	64.0	24.7		0.897	0.125	
Northwest	151	11.1	62.2	20.6		0.790	0.121	
Central	133	9.8	72.3	12.8		0.871	0.120	
Southwest	100	7.4	57.3	22.2		0.790	0.111	
South	135	9.9	66.1	18.6		0.823	0.134	
East	339	25.0	64.5	21.2		0.835	0.120	
Setting								
Urban	1,020	75.1	63.2	22.4	< 0.001	0.837	0.126	< 0.005
Rural	338	24.9	71.0	19.6		0.862	0.137	
Be married / living together								
Yes	941	69.3	65.0	22.2	> 0.05	0.844	0.130	> 0.05
No	417	30.7	65.5	21.6		0.843	0.128	
Education								
Middle school or lower	408	30.0	66.3	20.7	> 0.05	0.842	0.132	> 0.05
High school	499	36.7	66.1	22.0		0.843	0.130	
College or above	451	33.2	63.2	23.1		0.845	0.127	
Monthly income (Chinese Yuan) *								
< 1000	364	26.8	63.5	22.4	> 0.05	0.827	0.133	< 0.005
1000~	422	31.1	66.8	21.3		0.838	0.124	
2000~	223	16.4	65.8	22.8		0.852	0.131	
3000~	347	25.6	64.5	22.0		0.860	0.128	
Any insurance coverage*								
Yes	432	31.8	65.8	21.8	> 0.05	0.842	0.129	> 0.05
No	925	68.1	65.0	22.0		0.844	0.131	
Frequently smoking								
Yes	301	22.2	68.2	21.2	< 0.01	0.860	0.131	< 0.05
No	1,057	77.8	64.3	22.2		0.839	0.129	
Number of lifetime sexual partners*								
1	612	45.1	63.8	22.7	> 0.05	0.852	0.132	> 0.05
2	318	23.4	65.3	21.9		0.839	0.128	
3 or more	421	31.0	67.0	21.0		0.834	0.126	
Clinical status								
First clinical visit for the initially occurred GW	1,032	76.0	65.7	21.8	> 0.05	0.853	0.129	< 0.001
Follow-up visit for the initially occurred GW	146	10.8	64.1	24.4		0.822	0.126	
Recurrent GW	180	13.3	63.9	21.0		0.804	0.124	
Single or multiple genital warts								
Single	356	26.2	68.0	21.2	< 0.01	0.869	0.127	< 0.001
Multiple	1,002	73.8	64.2	22.2		0.835	0.129	

* Some data missing; ** Japanese preference weights applied; ^# ^One-way ANOVA analysis.

In the multivariate linear regression analysis, similar results were observed (Table 4); when using the VAS mean as the dependent variable, the variables of gender, rural or urban, residence in the North or Central regions of China, and single/multiple GW were retained in the final model (all *P *values < 0.01, adjusted R square = 0.065). When placing EQ-5D index score as the dependent variable, more factors entered the model, including initially occurred GW, monthly income, region of East, and region of Northeast (all *P *values < 0.001, adjusted R square = 0.117) (Table 4). The factors of smoking and monthly income were not statistically associated with the HRQoL of GW patients in the multivariate analysis.

**Table 4:** Mean VAS and EQ-5D index score, by population characteristics, using a multivariate linear regression approach


**Variable**	**Variable definition**	**Unstandardized Coefficients**		**Standardized Coefficients**	**t**	**Sig**.
				
		**B**	**SD**	**Beta**		

VAS mean						

Constant		63.721	1.455		43.795	0.000

Sex	Woman = 0; Man = 1	7.451	1.176	0.168	6.335	0.000

Setting	Rural = 0; Urban = 1	-6.459	1.361	-0.127	-4.747	0.000

Single/Multiple GW	Multiple GW = 0; Single GW = 1	4.138	1.334	0.082	3.101	0.002

Region-Central	Southwest = 0; Central = 1	8.653	2.042	0.117	4.237	0.000

Region-North	Southwest = 0; North = 1	3.655	1.341	0.074	2.725	0.007

Region-East	Southwest = 0; East = 1	0.032	0.010	0.108	3.145	0.002

EQ-5D index score*						

Constant		0.748	0.012		61.166	0.000

Sex	Woman = 0; Man = 1	0.035	0.007	0.134	5.140	0.000

Setting	Rural = 0; Urban = 1	-0.032	0.008	-0.106	-3.933	0.000

Single/Multiple GW	Multiple GW = 0; Single GW = 1	0.029	0.008	0.098	3.749	0.000

Region-Central	Southwest = 0; Central = 1	0.067	0.013	0.153	5.332	0.000

Region-North	Southwest = 0; North = 1	0.069	0.009	0.238	7.813	0.000

Region-East	Southwest = 0; East = 1	0.038	0.009	0.127	4.188	0.000

Region-Northeast	Southwest = 0; Northeast = 1	0.087	0.013	0.196	6.905	0.000

Clinical status- Recurrent GW	Recurrent GW = 0; Initially occurred GW = 1	0.031	0.008	0.103	3.968	0.000

Monthly income (Chinese Yuan)	< 1000 = 1; 1000 ~ = 2; 2000 ~ = 3; 3000 ~ = 4	0.012	0.003	0.107	4.040	0.000

* Japanese preference weights applied.

## Discussion

The current work is a unique large-scale multi-centre study focusing on the HRQoL of male and female patients with GW using a generic and internationally comparable instrument, the EQ-5D. We found that anxiety and depression posed a major problem for GW patients, and the measurement of VAS suggested that the HRQoL of GW patients was substantially lowered. The current analysis using different preference weights provided a range of utility estimates (overall and characteristic-specific) for future detailed QALY-related cost-effectiveness evaluations. This study also determined the characteristics of patents with relatively lower quality of life, including being female, living in urban areas and suffering multiple GWs, which will be informative for future GW prevention and control efforts.

The research group has published a parallel work conducted as another part of the whole GW project [5]. Both the studies explored the quality of life issue but from different perspectives and each study had its own emphasis. For example, firstly, the current study focused on both males and females, while the previous work mainly assessed female populations. Secondly, the current study was very focused on GW patients only, but the study by Wang et al included women who have a spectrum of HPV-related health statuses or laboratory diagnosis, including normal/abnormal pap smear, cervical precancer, and HPV+/- after abnormal pap, and patients with GW were only one sub-group in the broad analysis [5]. Thirdly, the previous work used a newly-developed HPV-specific instrument, the HPV impact profile (HIP); it is more sensitive and can recognize some slight differences among the targeted HPV-related subgroup women, but it is more difficult to directly compare the findings of the HIP study and other quality of life studies using different instruments. Differently, the EQ-5D, used by the current analysis, is an easy-to-compare HRQoL instrument and commonly-used world widely [12]. Finally, compared to the HIP score outcomes, the EQ-5D index score outcomes reported by the current study were relatively more convertible when taking qualifying quality-adjusted life year (QALY) saved as the study outcome.

The current analysis found that more than half of the GW patients in this survey were suffering anxiety and depression. This is a dramatically high proportion, when compared to the value of < 7% of the sampled general population (aged 15-49 years, by 5-year age group) across mainland China, as a part of the Chinese National Health Services Survey in 2008 (N = 120,703) [15]; and the proportion is also higher than that of some other countries (~ < 30%, pooled in a previous study by Wang et al) [17] and a group of GW patients in the UK (24%) [9]. This situation is due mainly to the relatively conservative culture and attitude to sex in China; being diagnosed with sexually transmitted diseases such as GW could be regarded as a big humiliation for patients and they usually would not let other people know and would not receive support even from their families. As expected, we also found that a high proportion of study cases were feeling pain and discomfort (24.7%) when compared to the Chinese general population ( < 10%, in patients aged 15-49 years) [15]. Providing questionnaire interview to part of the patients after their treatment could increase the feeling of pain, but the current study did not distinguish the ordering of treatment and interview. However, discomfort is commonly feeling in GW patients, which could explain some of this detriment. In contrast to the dimension of Anxiety/Depression, the dimensions of Mobility, Self-care and Usual Activities in this GW population are generally less impacted when compared to local and international general populations [15,17]; this situation is due partly to the reality that most of the studied GW patients (~93%) were younger than 50 years old, and were able to move, to take care of themselves, and to complete their usual activities with no difficulty. Also, the situation of lower rates of any problems in the three dimensions mentioned above is consistent with the findings of a UK GW study [9].

A large-scale survey included EQ-5D instrument was previously conducted based on a national representative sample in China in 2008, where the average VAS were found to be 80.9 for male and 79.4 for female [15]. Since HRQoL scores are very age-dependent but the subjects of current study mainly aged 15-49 years, we restricted the comparison to narrower age groups (15-49 years, by 5-year age group); it was noted that the national survey reported relatively higher values of VAS score (81.4-89.8 for male, 79.2-89.6 for female). The finding that the mean VAS scores in the current study are lower than those of Chinese general population (65.2 versus ~80 [15]) suggests that the HRQoL of patients with GW is notably lower than that of the general population. When compared to prior GW studies using EQ-5D in other countries, the mean VAS scores of the current Chinese GW cohort are lower than the estimate of an Australian study (68.9, N = 40) and of a UK study (72, N = 81) [6,9], but somewhat closer to the estimate of a Canadian study (65.1, N = 39) [11]. The observed differences between these GW cohorts in these settings might be explained by the differences in the VAS scores of the general populations behind them, which are 82.5, 78.7 and 80.1 for the UK, Canada and China, respectively. Based on results from other published works [9,15,17], it is not surprising to see that female GW patients had lower VAS scores than male patients. Although a VAS score declining with age was observed in a general population-based survey [15], our age curve of VAS scores shows a flat pattern, which is consistent with a prior UK GW study [9]. A potential reason for this inconsistency is that the majority of the GW patients were sexually active, and they might have a lower probability of susceptibility to a range of ageing diseases or situations.

Due to the absence of an EQ-5D preference weight set in the Chinese population, three other populations' preference sets were applied to estimate the EQ-5D index scores in the current analysis. Our findings suggest that scores based on the Japanese preference weights could be regarded as the baseline utility values for future cost-effectiveness evaluations, whilst the results based on the UK and US populations could provide a plausible range of utilities estimates for sensitivity analysis. When comparing our EQ-5D index scores to other international GW studies, a wide difference was noted but all our EQ-5D index scores using varied populations preference weights (0.826, 0.843 and 0.859) are within the range (0.76 - 0.91) of the available data from other populations [9-11]; the lowest utility value (0.76) of GW patients was from a Canadian study, and the highest value (0.91) was reported by Myers et al (conference abstract, details unavailable) which has been cited by a number of more recent cost-effectiveness evaluations of quadrivalent HPV vaccine [24-27]. Our EQ-5D index scores analysis also further supports the hypothesis that female patients suffered a larger decrease in quality of life than males and the scores did not differ notably among age groups, as we previously discussed in the VAS scores analysis.

In addition to the variable of gender, other characteristics could also potentially affect the HRQoL of GW patients. Our finding that the urban patients suffered a heavier physiological burden than rural patients could be explained by the urban residents' higher stress from job and mortgage payments and living a faster-paced life. This finding is identical with the results of another HRQoL analysis of HPV-related lesions (including GW) using a HPV-sensitive instrument [5]. It is not surprising to observe that subjects in Southwest China had the lowest score, because a prior survey (N = 2,830) has reported that the general population in Guizhou Province in the Southwest region of China had a relatively low HRQoL (VAS: ~68 - ~ 80 in residents aged 15-49 years) [18]. It is also understandable that more patients with multiple genital warts reported lower scores than patients with only single GW. Although most of the differences detected between subgroups were marginally less than the mean of clinically important differences in EQ-5D (0.074) [28], they were still within the range (0.011-0.140) [28], and could potentially generate a relatively significant impact on mass public health intervention programs. As for other variables marginally associated with the HRQoL of GW patients, including initial or recurrent GW, smoking, patient's monthly income and education level, they are beyond the scope of the current discussion, and more research needs to be done.

This analysis has some limitations. Firstly, selection biases could occur in this study, due to the convenient sampling approach we used; we also failed to collect information of non-attended patients and thus could not assess the differences in characteristics between the study participants and those who declined to participate. Secondly, use of the generic EQ-5D instrument, which is not sensitive to HPV-related diseases, potentially underestimates the negative impact from GW and a ceiling effect could occur. Thirdly, the current study is a questionnaire interview-based survey which is usually sensitive to the capacities of the interviewers and quality control, and some of the clinical physicians who administrated the interview had not directly received training provided by CICAMS, and quality-control could potentially vary among study centers to some extent. Another limitation of the study is that we did not have Chinese population specific preference weights and thus could not calculate the EQ-5D index scores accurately. Furthermore, combined with further data related to average duration of a clinical episode and the frequency of recurrence of GW, the detailed utilities findings from the current study would be informative for future cost-effectiveness evaluations related to quality-adjusted life years saved by new interventions against GW.

## Conclusions

The HRQoL of patients with GW was substantially lowered when compared to the general population in mainland China, and the quality of life was even worse among some sub-groups. It is vital to strengthen the public genital warts prevention program. In the era of HPV vaccination, although prophylactic HPV vaccines are not yet licensed in China, introducing a prophylactic vaccine protecting against HPV-6 and -11 would be feasible. Regarding the psychological effect of such an intervention on GW patients, effectively reducing patients' anxiety and depression can have a remarkably positive impact on the overall quality of life of GW patients. In addition, particular attention should probably be given to female patients and patients from urban areas and who are suffering worse clinical situations in clinical practice.

## Abbreviations

CFC: Cancer Foundation of China; CICAMS: Cancer Institute of Chinese Academy of Medical Sciences; EQ-5D: European quality of life-5 dimension; HRQoL: Health-related quality of life; GW: Genital warts; HPV: Human Papillomavirus; VAS: Visual analogue scale.

## Competing interests

The authors declare that they have no competing interests.

## Authors' contributions

YLQ was the Project Investigator and supervised the conduct of the study. YLQ and JFS contributed to the study design. DJK, SZQ, HYW, YCL, LJS, LL, YY, QL, XXF, LQZ, JL, XLL, YY, MN, ADX, JHL, XQ, LKL and XZW collected the field data. JFS performed all the data analyses with input and comments from YLQ. JFS wrote the first draft of the paper with input and comments from all other authors. All authors read and approved the final manuscript.

## Pre-publication history

The pre-publication history for this paper can be accessed here:

http://www.biomedcentral.com/1471-2458/12/153/prepub
